# Electronic and solvent effects on kinetics of S_N_Ar substitution reactions of substituted anilines with 2,6-bis(trifluoromethanesulfonyl)-4-nitroanisole in MeOH–Me_2_SO mixtures of varying composition: one reaction with two mechanistic pathways

**DOI:** 10.1007/s00706-013-1030-7

**Published:** 2013-07-12

**Authors:** Nizar El Guesmi, Guillaume Berionni, Basim H. Asghar

**Affiliations:** 1 Département de chimie, Faculté des Sciences de Monastir, 5019, Avenue de l’Environnement, Monastir, Tunisia; 2 UMR 8180, Institut Lavoisier-Franklin, Université de Versailles, 45, Avenue des Etats-Unis, 78035 Versailles Cedex, France; 3 Department of Chemistry, Faculty of Applied Sciences, Umm Alqura University, P.O. Box 9569, Makkah, Saudi Arabia

**Keywords:** Kinetics, Solvent effect, Solvatochromic parameters, Single electron transfer (SET) pathway, Biphasic concave upward free energy relationship

## Abstract

**Abstract:**

The kinetics and mechanism of the aromatic nucleophilic substitution reactions of 2,6-bis(trifluoromethanesulfonyl)-4-nitroanisole with *para*-X-substituted anilines (X = OH, OMe, Me, H, F, I, Cl) were studied in MeOH–Me_2_SO mixtures and pure Me_2_SO at 25.0 °C. The second-order rate coefficients depend on the substitutent in aniline and give good Hammett and Brønsted correlations; a polar S_N_Ar reaction is proposed for the reaction in different MeOH–Me_2_SO mixtures. The measured rate coefficients of the reaction demonstrated dramatic variations for aniline donor with the increasing dimethyl sulfoxide composition in MeOH–Me_2_SO mixtures. In this case, the Hammett and Brønsted plots are biphasic and concave upwards with a break point at 4-methylaniline. These results indicate a change in mechanism from the polar (S_N_Ar) for less basic nucleophiles (X = 4-Cl, 4-I, 4-F, and H) to the single electron transfer (SET) for more basic nucleophiles (X = 4-OH, 4-OMe and 4-Me). The changes of the structure of the transitions states with substituents and solvent are in accordance with the results of kinetics studies. The solvation model described is well supported by the solvatochromism exhibited by aniline in the solvent mixture under investigation. These results provide an ideal framework for understanding the paramount importance of the specific molecular structure of solvent molecules in determining chemical reactivity versus solvent effects.

**Graphical abstract:**

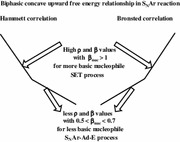

**Electronic supplementary material:**

The online version of this article (doi:10.1007/s00706-013-1030-7) contains supplementary material, which is available to authorized users.

## Introduction

Aromatic nucleophilic susbstitution reactions involving primary amines are an important class of organic synthetic reactions and continue to inspire studies of kinetics and mechanisms [1–5]. Studies have revealed that the displacement of the substituent at the 1-position is faster when the aromatic ring contains electron-withdrawing substituents such as –NO_2_, –CN, –CF_3_, or –SO_2_CF_3_ at *ortho* and *para* positions [1, 2, 6–10]. It is believed that this reaction generally proceeds through an addition–elimination mechanism. In the first step the nucleophile preferably attacks the position* ipso* to the leaving group of the electron-deficient aromatic ring to yield a zwitterionic intermediate. Typically, this intermediate with a tetrahedral (sp^3^) carbon is unstable, and the reaction could proceed forward by rearomatization to generate the substituted product (Scheme 1).

**Figure:**
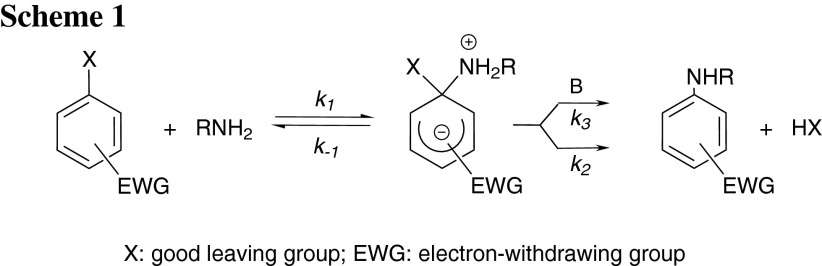

The reactivity of aromatic nucleophilic substitution (S_N_Ar) reactions has been extensively investigated, and is notably affected by the solvent. The role of the solvent in governing a chemical reaction is far from passive. Therefore, a proper understanding of solvent effects is essential to any model of chemical reactivity [11–13]. In addition, others factors such as the effect of the aromatic ring substituents, ring size of the nucleophile, and the electronic nature and position of the substituents affect the rate of the aromatic nucleophilic substitution reactions [14–18]. Some of the factors that affect the reaction rates are closely related to the nature and extent of solute–solvent interactions (the solvation effect) locally developed in the immediate vicinity of the solute, solvent–solvent interactions (the general medium effect), and solute–solute interactions (the intersolute effects) [19–22]. The study of solute–solvent interactions in binary mixtures is more complex than in pure solvents as a result of preferential solvation [23].

Most of the previous studies on solvent effects in S_N_Ar have been performed in pure solvents. Nevertheless, different studies aimed at the characterization of mixed solvents and the determination of the kinetic data of S_N_Ar reactions in binary mixtures have been recently reported [24–26].

Examination of the literature revealed that the effects of structure on S_N_Ar reactions have largely been reported [27–36]. However, only very few attempts have been made to study the effect of solvent on such reactions in a more systematic manner [25, 26, 37, 38].

The study of the influence of the solvent on the reactions of anilines in nonaqueous solvent mixtures has revealed the important role of nonspecific and specific solvent effects on reactivity [39, 40]. Chemists have usually attempted to understand such interactions in terms of “solvent polarity”, which was used synonymously with the power to solvate solute charges. It was assumed to increase with the dipole moment of the solvent molecules and to decrease with increased thickness of shielding of the dipole charges [22, 23]. Therefore, mechanistic study on the nucleophilic substitution reaction between aromatic compounds having strong electron-withdrawing substituents and aromatic amines is very important in examining the effects of dipolar protic and aprotic solvents in the ground state or transition state.

The significance of structure–reactivity relationships based on the parameters of mechanistic criteria such as Hammett *ρ* value and Brønsted coefficients for nucleophilic substitution or addition reactions has been discussed by Jencks [41]. The *β*_nuc_ values are commonly accepted as measures of the degree of charge transfer, from the nucleophile to the electrophile partner, at the transition state (TS) [41]. In that sense, it could be anticipated that the normal range of *β*_nuc_ values would be between 0 and 1. However, it was discovered through the work of Bordwell, Jencks, Bernasconi, and others [42–46] that certain processes were characterized by *β* values out of this normal range. Most of the S_N_2 reactions are characterized by *β*_nuc_ values in the 0.2–0.5 range [42, 43]. However, *β*_nuc_ values close to or greater than 1.0 have been observed for other S_N_2-type reactions of carbanions and nitranions with sulfonyl- and nitro-activated aromatic halides. These results were interpreted as indicative of the occurrence of complete electron transfer [47]. Regarding S_N_Ar reactions, Bordwell, on the basis of the numerous available results of *β*_nuc_ values falling in the range 0.5–0.7, emphasized that these reactions entail a relatively large transfer of electronic charge in the TS [47, 48]. In contrast, few examples of *β*_nuc_ values are greater than 1 [46], and these results may be regarded as indicative of the incursion of a single electron transfer (SET)-type mechanism.

This subdivision of nucleophilic substitution reactions into either polar or SET pathways is equally applicable to all the other fundamental organic mechanisms. Thus hydride reduction, electrophilic and nucleophilic aromatic substitution, and nucleophilic addition, to quote a few examples, may also be formulated in terms of either SET or polar mechanisms. In view of the existing uncertainty, a number of questions arise: (a) What are the factors that determine whether a particular reaction proceeds via SET or a polar pathway? (b) What is the precise relationship between the two possible processes?

Hence, in continuation of studies in the field of S_N_Ar reactions [49], we report herein the investigation of the solvent effect on the kinetic of reaction of *para*-substituted anilines with 2,6-bis(trifluoromethanesulfonyl)-4-nitroanisole in methanol (MeOH)/dimethyl sulfoxide (Me_2_SO) mixtures of varying composition. In addition, the study applies structure–reactivity correlations as a useful diagnostic tool to understand the quantitative solvent effect on the rate and mechanism of the reaction.

## Results and discussion

The kinetic study was performed under pseudo-first-order conditions with the concentration of anilines in excess over the substrate concentration. All of the reactions obeyed first-order kinetics. Pseudo-first-order rate constants (*k*_obs_) were calculated from the equation ln $(A_{\infty } - A_{\text{t}} )= - k_{\text{obs}} t + C$. The *k*_obs_ values and the reaction conditions are summarized in Tables S1–S7 in the Supplementary Material.

The pseudo-first-order rate constants observed (*k*_obs_) for all reactions obey Eq. (1) with negligible *k*_o_ (≈0) in MeOH–Me_2_SO mixtures (Fig. S1–S7 in the Supplementary Material). The second-order rate constants *k*_1_ were determined using Eq. (1), no third-order or higher-order terms were detected, and no complications were found in the determination of *k*_obs_ or in the linear plot of Eq. (1).

1 $$ k_{\text{obs}} = k_{ 0} + k_{ 1} [{\text{An}}] $$

This suggests that there is no base catalysis or noticeable side reactions, and the overall reaction follows the route given by Scheme 2. The second-order rate constants *k*_1_ of the anilinolysis of 2,6-bis(trifluoromethanesulfonyl)-4-nitroanisole (**1**) at 25 °C in MeOH–Me_2_SO mixtures are summarized in Table 1. The substituent effects of the nucleophiles on the rates are in accordance with those for a typical nucleophilic substitution reaction, i.e., a stronger nucleophile results in a faster rate. As shown in Table 1, the second-order rate constant increases as the substituent X changes from an electron-withdrawing group (EWG) to an electron-donating group (EDG).

**Figure:**
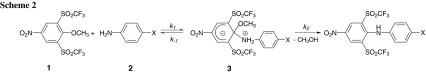

**Table 1:** Second-order coefficients (*k*_1_/mol^−1^ dm^3^ s^−1^) for the reaction of anilines with 2,6-bis(trifluoromethanesulfonyl)-4-nitroanisole in various vol% of dimethyl sulfoxide (Me_2_SO) in methanol (MeOH) at 25 °C

Aniline substitutent (10^3^ × *k*_1_)	Me_2_SO/vol%						
	0	10	30	50	70	90	100
None	5.01^a^	5.46	6.89	10.1	12.0	22.4	31.6
*p*-OH	20.0^b^	22.5	35.1	43.6	56.2	162.0	289.0
*p*-OMe	11.7^a^	16.3	25.0	33.3	43.3	76.6	144.0
*p*-Me	8.14^a^	9.84	14.3	16.6	21.6	36.1	58.7
*p*-F	4.68^a^	5.31	6.66	9.40	11.2	21.4	27.8
*p*-I	2.63^b^	3.09	3.76	5.13	6.46	11.2	15.0
*p*-Cl	2.03^a^	2.43	3.05	4.07	5.36	10.2	12.8

^a^ *k*_1_ values in pure methanol were taken from [49]

^b^ *k*_1_ values determined in this work

**Fig. 1:**
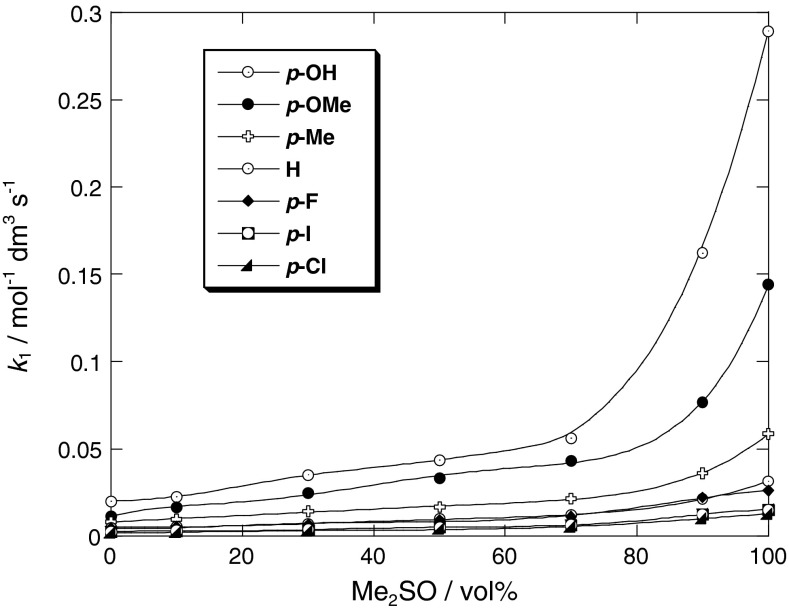
Plots of *k*_1_ versus vol% of Me_2_SO in MeOH–Me_2_SO mixtures for the reaction of 2,6-bis(trifluoromethanesulfonyl)-4-nitroanisole with *para*-substituted anilines at 25 °C

Table 1 also shows that the second-order rate constant (*k*_1_) for the reaction of **1** with anilines **2** increases with increasing the dimethyl sulfoxide volume percent, i.e., increases from 20.0 × 10^−3^ mol^−1^ dm^3^ s^−1^ in methanol to 289 × 10^−3^ mol^−1^ dm^3^ s^−1^ in Me_2_SO for X = 4-OH. Figure 1 shows a plot of the reaction rate constant versus volume percent of Me_2_SO. As can be seen, the rate constant of the reaction increases sharply with the Me_2_SO content. Although changes in the overall reactivity with the variation of substituent X in aniline show a similar tendency in all MeOH–Me_2_SO mixtures, the rate enhancement due to the variation of substituent X, i.e., *k*_4-OH_/*k*_4-H_ and *k*_4-OMe_/*k*_4-H_, in Me_2_SO is greater than in methanol solvent: the values are 9.15 and 4.56, respectively, in Me_2_SO, whereas the values are 4.00 and 2.33, respectively, in MeOH. This may be attributed to the reduced nucleophilicity of substituted anilines in methanol solvent because of the hydrogen bond between nucleophiles (anilines) and methanol molecules. It is noted that aniline hydrogen bonded by methanol is less reactive than free aniline in Me_2_SO solvent: the attacking aniline is a weak nucleophile in methanol, but becomes more reactive in Me_2_SO. The decrease in the second-order rate constant (*k*_1_) by increasing the volume percent of methanol indicates that the ground state (GS) stabilization energy due to the hydrogen bond with methanol solvent is larger than that of transition state (TS), because the nitrogen atom of aniline can conjugate with the aromatic ring and the hydrogen bond between the solvent (methanol) and the aniline is weaker.

### Solvent effect

In order to interpret the influence of the solvent effects on the explored S_N_Ar reaction, we performed a correlation analysis between the kinetic data and the molecular microscopic solvent properties. In order to determine the incidence of each type of solvent property on the kinetics of the reaction, we applied a quantitative treatment of the solvent effects by means of the multiparametric approach developed by Kamlet, Abboud, and Taft (KAT) [50, 51]. The KAT equation contains nonspecific as well as specific solute–solvent interactions separately. In general, these parameters constitute more comprehensive measures of solvent polarity than the dielectric constant alone, because they reflect more reliably the complete picture of all intermolecular forces acting between solute and solvent molecules. This approach has been widely and successfully applied in the correlation analysis of all kinds of solvent-dependent processes [52–56]. Using the solvatochromic parameters π*, *α*, and *β*, where π* is the index of the solvent dipolarity/polarizability, which is a measure of the ability of a solvent to stabilize a charge or a dipole by its own dielectric effects. The *β* coefficient represents the solvent hydrogen bond donor (HBD) acidity; in other words, it describes the ability of a solvent to donate a proton in a solvent to a solute hydrogen bond. The *β* coefficient is a measure of solvent hydrogen bond acceptor (HBA) basicity and describes the ability of a solvent to accept a proton in a solute to solvent hydrogen bond.

In this work, we have also used the polarity scale proposed by Dimroth and Reichardt, *E*_T_ [23, 57, 58], this scale has now been revised and normalized to $E_{T}^{\text{N}}$, known as the normalized polarity parameter, due to the introduction of SI units. $E_{T}^{\text{N}}$ is related to the ability of a solvent to stabilize charge separation. The KAT and $E_{T}^{\text{N}}$ parameters for all of the MeOH–Me_2_SO mixtures are listed in Table 2.

**Table 2:** Solvent parameters in mixtures of methanol and dimethyl sulfoxide at 25 °C

Me_2_SO/vol%	Solvatochromic parameter			
	$E_{T}^{\text{N}}$ ^a^	π*^b^	*α* ^b^	*β* ^b^
0	0.755	0.586	0.980	0.620
10	0.751	0.652	0.704	0.641
30	0.732	0.756	0.297	0.641
50	0.700	0.826	0.184	0.702
70	0.640	0.893	0.079	0.737
90	0.540	0.963	0.010	0.757
100	0.442	1.00	0.00	0.764

$E_{T}^{\text{N}}$, π*, *α*, and *β* are normalized polarity parameter, dipolarity/polarizability, hydrogen bond donor, and hydrogen bond acceptor abilities of the solvent, respectively

^a^Values taken from [59]

^b^Values taken from [60]

**Fig. 2:**
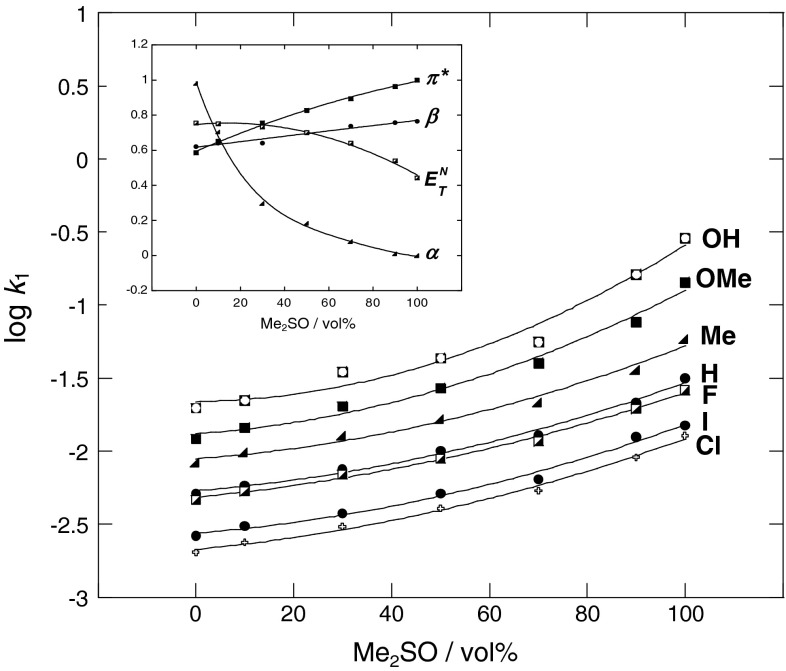
Plots showing dependence of log *k*_1_ on variation of solvatochromic π*, *α*, *β*, and $E_{T}^{\text{N}}$ parameters with vol% of Me_2_SO in MeOH–Me_2_SO mixtures in reaction of 2,6-bis(trifluoromethanesulfonyl)-4-nitroanisole with *para*-substituted anilines at 25 °C

As can be seen, the reaction rate constant increases with π* and *β* parameters and decreases with *α*; this behavior is illustrated in Fig. 2. The intermediate of the reaction has zwitterionic character (Scheme 2), and the activated complex of the reaction, therefore, has higher polarity relative to those of the reactants. The activated complex with zwitterionic character is expected to be favored by the increase in the $E_{T}^{\text{N}}$ and π* of media, because zwitterionic molecules were more stabilized in higher polarity media than in lower polarity media; but, in this case the rate reaction decreases with the increase in the $E_{T}^{\text{N}}$ of the media. Hence, it is evident that the polarity of the solvent does not suffice to explain the experimental observations.

Normally, the presence of methanol decreases the reaction rate in this type of reaction because of its HBD character [15, 16]. In fact, the low basicity of the aniline derivatives would play a role in the solvent–nucleophile interactions. Contrary to the normalized polarity, the HBD ability of the solvent reduces the rate of reaction. Two reasons can be attributed for this reduction. Firstly, in the presence of aniline or its derivations, methanol is known to act as an HBD, and there is evidence of strong hydrogen-bonding interaction between anilines and methanol [55]. Therefore, anilines are stabilized via this interaction, and the reaction rate decreases as the HBD ability of the media increases. Secondly, Me_2_SO is an HBA molecule, and methanol is an HBD species in the solvent mixtures. Strong solvent–solvent interactions in this media can be related to the hydrogen-bonding interaction between methanol and Me_2_SO to give a complex structure that is more or less polar than the two constituents of the mixture. This behavior is attributed to the preferential solvation of solutes by mixed solvent [20–22, 55].

The intermediate of the reaction has a positive charge on the nitrogen of aniline and a negative charge on the benzene ring. Then, hydrogen-bonding interactions of the media (solvent as acceptor with *β* parameter) with positive charge on the activated complex of the reaction will stabilize the activated complex better than the reactants; therefore, increasing the *β* parameter accelerates the reaction rate.

Hydrogen-bonding interactions of the media (solvent as donor with *α* parameter) with electron pairs will stabilize the reactant more than the activated complex of the reaction, because the negative charge of the activated complex of the reaction is distributed on the benzene ring, but the electron pair in aniline is mainly located on the nitrogen atom. Therefore, aniline will be stabilized via hydrogen-bonding interactions with hydrogen bond donors. For this reason the reaction rate constant decreases with *α* of the media.

Thus, increase in the mole fraction of Me_2_SO in the mixture progressively decreases the solvation around the NH_2_ moiety of the aniline molecule. Hence, the observed increase in rate of the reaction between aniline and 2,6-bis(trifluoromethanesulfonyl)-4-nitroanisole with increase in the mole fraction of Me_2_SO might be due to the desolvation of the NH_2_ moiety to a relatively greater extent. On the other hand, the second-order rate coefficients increase rapidly with the increasing mole fraction of Me_2_SO between aniline donor (X = OH and OMe) and 2,6-bis(trifluoromethanesulfonyl)-4-nitroanisole; this behavior might also be due to the desolvation of the X moiety of aniline.

The solvation effects are dominated by the nonspecific interactions. The rate constant is more influenced by the solvent effects attributed to dipole and induced-dipole interactions than those due to the hydrogen bond interactions. Moreover, the incidence of the solvation effects ascribed to the HBA solvent properties are more important than those corresponding to the HBD solvent character.

### Effect of substituent on reaction mechanism

**Fig. 3:**
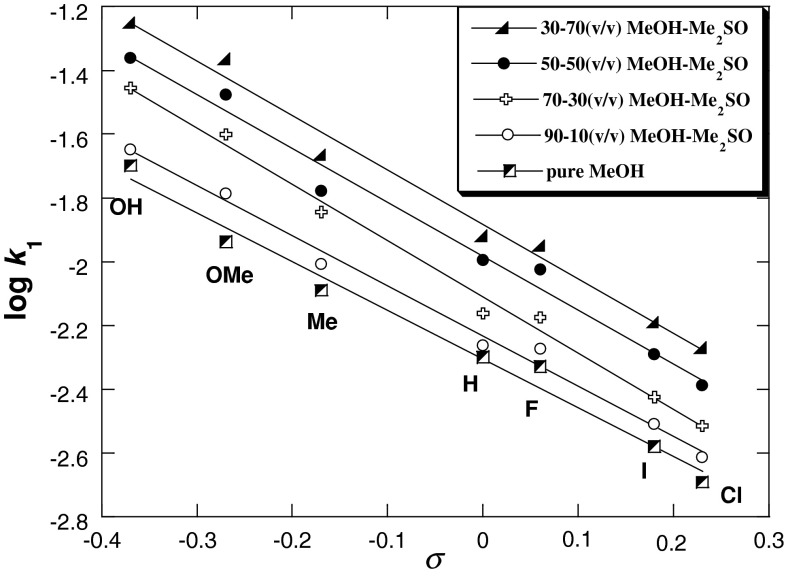
Hammett plots of the anilinolysis of 2,6-bis(trifluoromethanesulfonyl)-4-nitroanisole in pure methanol and 90:10, 70:30, 50:50, and 30:70 (v/v) MeOH–Me_2_SO at 25 °C

**Fig. 4:**
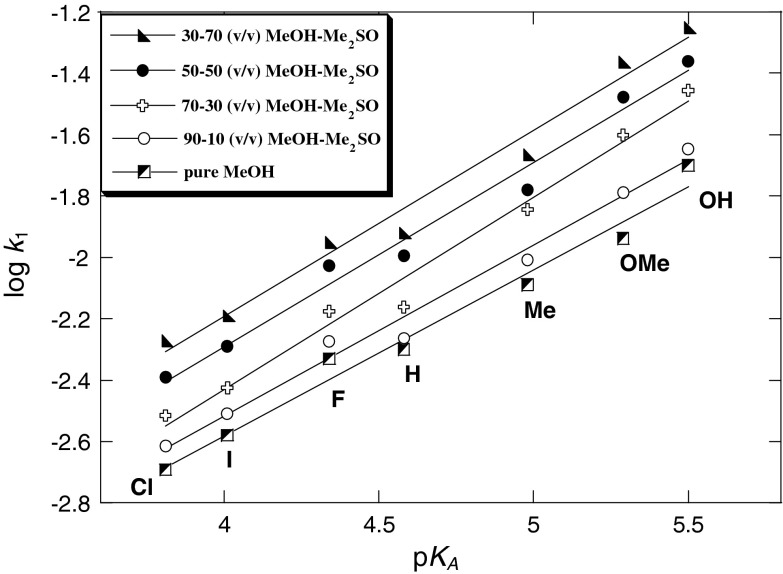
Brønsted plots of the anilinolysis of 2,6-bis(trifluoromethanesulfonyl)-4-nitroanisole in pure methanol and 90:10, 70:30, 50:50, and 30:70 (v/v) MeOH–Me_2_SO at 25 °C

**Fig. 5:**
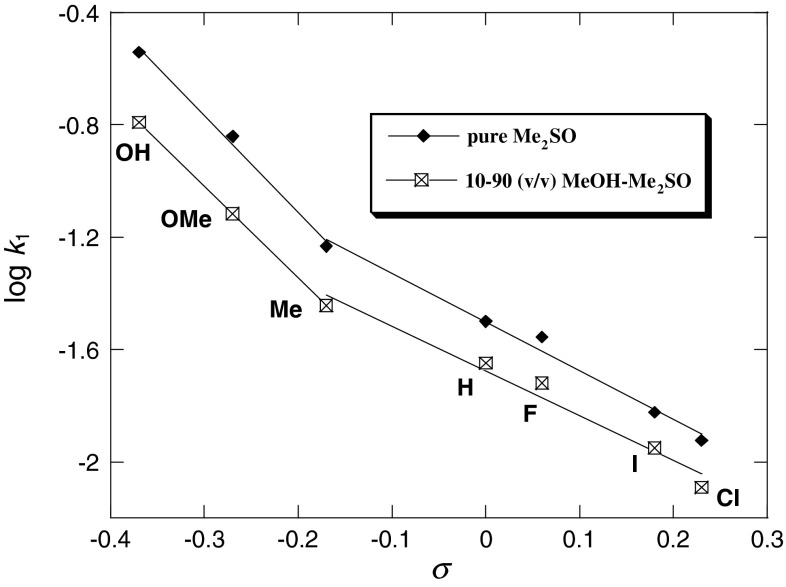
Hammett plots of the anilinolysis of 2,6-bis(trifluoromethanesulfonyl)-4-nitroanisole in 10:90 (v/v) MeOH–Me_2_SO and pure Me_2_SO at 25 °C

**Fig. 6:**
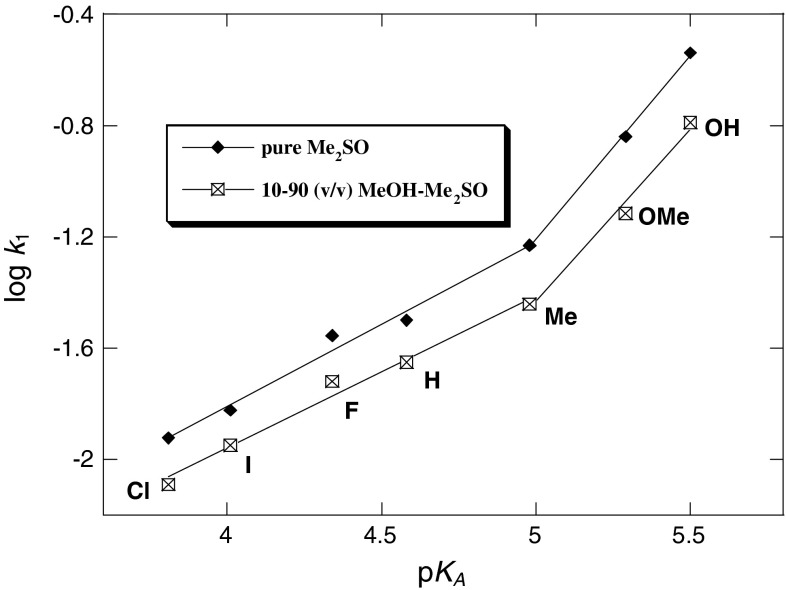
Brønsted plots of the anilinolysis of 2,6-bis(trifluoromethanesulfonyl)-4-nitroanisole in 10:90 (v/v) MeOH–Me_2_SO and pure Me_2_SO at 25 °C

Figures 3, 4, 5, and 6 show the Hammett and Brønsted plots for reactions of 2,6-bis(trifluoromethanesulfonyl)-4-nitroanisole with *para*-substituted anilines in pure methanol, 90:10, 70:30, 50:50, and 30:70 (v/v) MeOH–Me_2_SO mixtures and 10:90 (v/v) MeOH–Me_2_SO mixtures and pure Me_2_SO, respectively. Hammett *ρ*_X_ values obtained from the plots of log *k*_1_ versus *σ* for substituents on aniline are summarized in Table 3 together with *β*_X_ values determined from extended Brønsted treatments by plotting log *k*_1_ (MeOH–Me_2_SO) against p*K*_A_ (H_2_O) of anilines.

**Table 3:** Hammett *ρ*_X_ and Brønsted *β*_X_ coefficients of the anilinolysis of 2,6-bis(trifluoromethanesulfonyl)-4-nitroanisole in various vol% of dimethyl sulfoxide (Me_2_SO) in methanol (MeOH) at 25 °C

Hammett and Brønsted coefficients	Me_2_SO/vol%						
	0	10	30	50	70	90	100
*ρ*_X_	−1.53 (0.9922)^a^	−1.57 (0.9960)	−1.77 (0.9967)	−1.69 (0.9925)	−1.71 (0.9939)	−1.59^b^ (0.9859)	−1.68^b^ (0.9950)
						−3.26^c^ (0.9999)	−3.45^c^ (0.9971)
*β*_X_	0.55 (0.9896)	0.56 (0.9935)	0.62 (0.9927)	0.60 (0.9916)	0.61 (0.9908)	0.54^b^ (0.9922)	0.59^b^ (0.9928)
						1.24^c^ (0.9940)	1.32^c^ (0.9994)

*σ* values were taken from [69]. p*K*_A_ values in water were taken from [70]

^a^Correlation coefficient (*r*)

^b^X = 4-Me and 4-Cl

^c^X = 4-OH and 4-Me

Figures 3 and 4 yield the linear free energy correlations, whereas the Hammett and Brønsted plots for substituent X variations in Figs. 5 and 6 are biphasic and concave upwards with a break point at 4-Me-aniline. In the Hammett plots, the magnitudes of *ρ*_X_ and *β*_X_ of strongly basic anilines (X = 4-OH, 4-OMe, and 4-Me) are greater than those of weakly basic anilines (X = 4-H, 4-F, 4-I, and 4-Cl). In general, in nucleophilic substitution reactions, a concave upward non-linear free energy correlation plot is diagnostic of a change in the reaction mechanism [61–65], such as parallel reactions where the reaction path is changed depending on the substituents, whereas a concave downward non-linear free energy correlation plot is diagnostic of a rate-limiting step change [64–68]. We suggest that the concave upward Hammett and Brønsted plots (Figs. 5, 6) can also be diagnostic of a change in the reaction mechanism depending on the substituents from polar to SET.

Table 3 shows that the transition parameter *ρ*_X_ values are −1.53, −1.57, −1.77, −1.69, and −1.71 in MeOH and 90:10, 70:30, 50:50, and 70:30 (v/v) MeOH–Me_2_SO, respectively. These results are comparable with the values (−1.75 < *ρ* < −1.98) reported by Sung for substitutions of 2,4,6-trinitrochlorobenzene by substituted pyridines in MeOH–MeCN mixtures [71], and are also similar to the results for the substitution reaction of 2-chloro-5-nitropyridine with *para*-substituted anilines in 70:30 (v/v) Me_2_SO–MeCN mixtures [72], and arenethiolates in methanol (*ρ* = −1.80) [73]. The *β*_X_ values are 0.55, 0.56, 0.62, 0.60, and 0.61 in MeOH and 90:10, 70:30, 50:50, and 70:30 (v/v) MeOH–Me_2_SO (Table 3), respectively; similar slopes were found in other reactions, such as 2,4-dinitro-1-fluorobenzene with alicyclic secondary amines in H_2_O (*β*_nuc_ = 0.52) [74] and 4-cyano-2,6-dinitrochlorobenzene with substituted pyridines in MeOH–MeCN mixtures (0.52 < *β*_nuc_ < 0.57) [71].

The negative *ρ*_X_ and positive *β*_X_ values obtained in the present work are consistent with significant development of a positive charge at the nitrogen atom of the aniline moiety and the negative charge developed in the substrate aromatic ring in the TS for formation of a zwitterionic intermediate σ complex. The zwitterionic intermediate (Meisenheimer σ complex) is stabilized through delocalization of negative charge by resonance, as shown in Scheme 3 [pathway (a)]. The aforementioned values are in keeping with the traditional interpretation of nucleophilic aromatic substitution by amines, and this behavior accords well with the S_N_Ar-Ad.E mechanism shown in Scheme 3 [pathway (a)], where rate-limiting formation of the intermediate σ complex is followed by fast expulsion of the methoxy leaving group.

**Figure:**
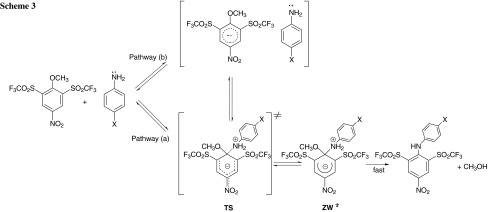

However, both the Hammett (log *k*_1_ versus *σ*_X_, Fig. 5) and Brønsted (log *k*_1_ versus p*K*_A_(X), Fig. 6) plots in a higher composition of Me_2_SO are biphasic and concave upwards with a break point at X = 4-Me. The magnitudes of the values of *ρ*_X_ and *β*_X_ in 10:90 (v/v) MeOH–Me_2_SO mixture (*ρ*_X_ = −3.26, *β*_X_ = 1.24) and pure Me_2_SO (*ρ*_X_ = −3.45, *β*_X_ = 1.32) with the strongly basic anilines (X = 4-OH, 4-OMe, 4-Me) are much greater than those in 10:90 (v/v) MeOH–Me_2_SO mixture (*ρ*_X_ = −1.42, *β*_X_ = 0.49) and pure Me_2_SO (*ρ*_X_ = −1.73, *β*_X_ = 0.59) with the weakly basic anilines (X = 4-Me, H, 4-F, 4-I, 4-Cl).

As seen in Table 3, the *β*_X_ values of 2,6-bis(trifluoromethanesulfonyl)-4-nitroanisole in pure Me_2_SO and in 10:90 (v/v) MeOH–Me_2_SO mixture with the weakly basic anilines are similar to those previously reported in various MeOH–Me_2_SO mixtures suggesting the same reaction mechanisms.

On the other hand, the large *β*_nuc_ values show greater sensitivity to substituent changes on the reaction at hand relative to the reference ionization equilibrium [46, 75–77], or in the case of S_N_2 reactions in terms of the advent of a SET pathway, where full electronic transfer occurs prior to the coupling of electrophilic and nucleophilic partners [47, 48]. The high *β*_X_ values associated with the present reactions may be a reflection of a SET pathway, as described in Scheme 3 [pathway (b)]. As in Scheme 3 [pathway (b)], one of the electrons of the lone pair in aniline (donor) is transferred to the 2,6-bis(trifluoromethanesulfonyl)-4-nitroanisole acceptor moiety, and subsequent coupling between the resulting cation and anion radicals within the solvent cage takes place. The transition state for the coupling reaction might be structure TS, and σ complex intermediate ZW will be formed as a result.

**Fig. 7:**
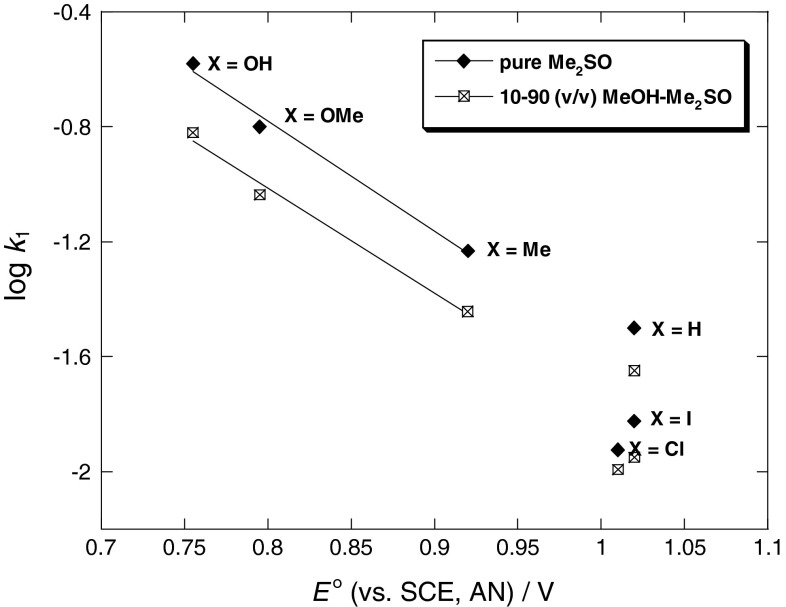
Influence of the oxidation potential *E* ^°^ of anilines on the rate of reaction of 2,6-bis(trifluoromethanesulfonyl)-4-nitroanisole with *para*-substituted anilines in pure Me_2_SO and 10:90 (v/v) MeOH–Me_2_SO mixture at 25 °C

One-electron reduction potentials *E* ^°^ of 4-X-anilines in aqueous solutions were measured by Jonsson et al. [75] and Bacon and Adams [76]. Both the *E* ^°^ versus *σ* ^+^ and p*K*_A_ versus *σ* ^+^ plots show good linearity [75–77]. This indicates that the *β*_X_ values are associated with one-electron reduction (or oxidation potential) *E* ^°^. Plots of log *k*_1_ against *E* ^°^ values of 4-substituted anilines show a good linear relationship with strongly basic anilines (X = OH, OMe, and Me), as indicated in Fig. 7. These results are clearly consistent with the SET pathway, as shown in Scheme 3 [pathway (b)].

## Conclusions

The kinetic studies of the reactions of 2,6-bis(trifluoromethanesulfonyl)-4-nitroanisole with substituted X-anilines have been carried out at 25.0 °C in 90:10, 70:30, 50:50, 30:70, 10:90, and 0–100 (v/v) MeOH–Me_2_SO. Changes in the solvent composition showed different effects on the rate of aromatic nucleophilic substitution reaction of 2,6-bis(trifluoromethanesulfonyl)-4-nitroanisole with anilines in methanol mixed with dimethyl sulfoxide. The analysis of the agreement of solvent property values obtained with comparable solutes reveals that the rate constant of the reaction increases with π* and *β* parameters and decreases with *α*. The results clearly demonstrate that, in the mixture of protic–aprotic solvents, formation of the zwitterionic intermediate is the rate-determining step of the reaction. Dipolarity/polarizability, hydrogen bond donor, and hydrogen bond acceptor abilities of the media have the strongest effects on the reaction rates.

The S_N_Ar reaction analyzed reflects two different kinetic response models depending on the nucleophile strength and as a function of the solvent composition. An S_N_Ar-Ad.E with nucleophilic attack is rate-limiting and formation of the intermediate σ complex followed by fast expulsion of the methoxy leaving group is proposed for the anilinolysis of 2,6-bis(trifluoromethanesulfonyl)-4-nitroanisole in 90:10, 70:30, 50:50, and 30:70 (v/v) MeOH–Me_2_SO mixtures. In the case of the anilinolysis of 2,6-bis(trifluoromethanesulfonyl)-4-nitroanisole in 10:90 (v/v) MeOH–Me_2_SO mixture and pure Me_2_SO, the Hammett and Brønsted plots are biphasic and concave upwards with a break point at 4-methylaniline indicating a change in mechanism from an S_N_Ar-Ad.E for less basic nucleophiles (X = 4-Cl, 4-I, 4-F, and H) to a SET process for more basic nucleophiles (X = 4-OH, 4-OMe and 4-Me). On the basis of the higher *β*_X_ values (1.24 and 1.32) of the reaction and a good correlation of the rate constants with the oxidation potentials for more basic nucleophiles (X = 4-OH, 4-OMe and 4-Me), the reaction was initiated by a SET mechanism, where one of the electrons in aniline is transferred to 2,6-bis(trifluoromethanesulfonyl)-4-nitroanisole. After this step, the reaction of 2,6-bis(trifluoromethanesulfonyl)-4-nitroanisole, which is an electrophilic benzenoide system, proceeds through a transition state similar to the normal S_N_Ar-Ad.E pathway.

To conclude, from the foregoing results and discussion, the concave upward free energy relationship can be diagnostic of a change in the mechanism in aromatic nucleophilic substitution reactions. In addition, our large *β*_X_ (1.24 and 1.32) values confirm the idea that an ‘abnormal’ *β*_nuc_ value may be an indicator of electron transfer in aromatic nucleophilic substitution reactions [46].

## Experimental

2,6-Bis(trifluoromethanesulfonyl)-4-nitroanisole was prepared as previously described by Boiko et al. [78]. Anilines were of the highest quality available and were recrystallized or distilled before use whenever necessary. They were commercial specimens (Aldrich products). Methanol was used without further purification. Dimethyl sulfoxide (Me_2_SO) was refluxed over calcium hydride and distilled, and the fractions boiling at 32–35 °C were collected and stored under nitrogen. All binary solvent mixtures were prepared prior to use and stored under anhydrous conditions.

### Rate measurements

Kinetic determinations were performed on an Applied Photophysics SX-18MV stopped-flow apparatus or a conventional Shimadzu (model 1650 PC) UV–Vis spectrophotometer, the cell compartments of which were maintained at 25 ± 0.1 °C. All kinetic runs were carried out in triplicate under pseudo-first-order conditions with a triflone concentration of ~5 × 10^−5^ mol dm^−3^ and an aniline concentration in the range of 5 × 10^−3^−0.1 mol dm^−3^. In a given experiment, the rates were found to be reproducible to 2–3 %.

## Electronic supplementary material

Below is the link to the electronic supplementary material.

Supplementary material 1 (DOC 402 kb)
