# Supplementary material for: Electronic and solvent effects on kinetics of SNAr substitution reactions of substituted anilines with 2,6-bis(trifluoromethanesulfonyl)-4-nitroanisole in MeOH–Me2SO mixtures of varying composition: one reaction with two mechanistic pathways
Source: Monatsh Chem. 2013 Jul 12;144(10):1537–45. doi: 10.1007/s00706-013-1030-7 (PMC4495024; doi:10.1007/s00706-013-1030-7)
Supplement: Supplementary file 1 — Supplementary material 1 (DOC 402 kb) [file 706_2013_1030_MOESM1_ESM.doc]

**Supplementary Materials**

**Table S1** Kinetic results for reaction of 2,6-bis(trifluoromethanesulfonyl)-4-nitroanisole with 4-hydroxyaniline in MeOH-Me_2_SO (v/v) mixtures at 25 °C.

| [4-hydroxyaniline]  / mol dm^-3^ | 10^4^ x k_obs_ / s^-1^ | | | | | |  |
| --- | --- | --- | --- | --- | --- | --- | --- |
|  | MeOH | MeOH-Me_2_SO (v/v) mixtures | | | | | *Me_2_SO* |
|  |  | 90-10 | 70-30 | 50-50 | 30-70 | 10-90 |  |
| 0.005 | 1.10 | 1.18 | 1.95 | 2.34 | 2.55 | 12.0 | - |
| 0.01 | 2.15 | 2.42 | 3.82 | 4.71 | 4.95 | 19.6 | 38.1 |
| 0.02 | 3.85 | 4.45 | 7.22 | 7.46 | 10.8 | 29.7 | 61.7 |
| 0.03 | 5.43 | - | - | 14.0 | 19.2 | 47.7 | 79.7 |
| 0.04 | 7.42 | 9.11 | 13.3 | 18.1 | 23.7 | 60.3 | 105.3 |
| 0.05 | 9.44 | 10.7 | 17.5 | 20.4 | 29.6 | 83.6 | 143.6 |
| 0.06 | 11.8 | 13.6 | 21.6 | 26.1 | 35.5 | 105.8 | 165.8 |
| 0.07 | 13.9 | - | - | - | - | 111.6 | 207.8 |
| 0.08 | 16.3 | 18.3 | 28.3 | 35.3 | 43.1 | 130.5 | 238.2 |

Correlation coefficients of the linear regressions were higher than 0.9944

**Table S2** Kinetic results for reaction of 2,6-bis(trifluoromethanesulfonyl)-4-nitroanisole with 4-methoxyaniline in MeOH-Me_2_SO (v/v) mixtures at 25 °C.

| [4-methoxyaniline]  / mol dm^-3^ | 10^4^ x k_obs_ / s^-1^ | | | | | |  |
| --- | --- | --- | --- | --- | --- | --- | --- |
|  | MeOH | MeOH-Me_2_SO (v/v) mixtures | | | | | Me_2_SO |
|  |  | 90-10 | 70-30 | 50-50 | 30-70 | 10-90 |  |
| 0.005 | 0.67 | 0.82 | 1.34 | 1.69 | - | - | - |
| 0.01 | 1.20 | 1.76 | 2.57 | 3.71 | 4.82 | 7.15 | 14.6 |
| 0.02 | 2.18 | 3.31 | 5.05 | 7.02 | 7.65 | 14.8 | 28.7 |
| 0.025 | 3.01 | - | - | - | - | - | - |
| 0.03 | 3.53 | - | - | - | 13.7 | 24.2 | 47.7 |
| 0.04 | 4.73 | 5.96 | 10.1 | 11.9 | 17.7 | 31.8 | 60.3 |
| 0.05 | - | 8.67 | 12.9 | 16.6 | 21.1 | 37.9 | 73.6 |
| 0.06 | 7.06 | 9.77 | 14.9 | 20.9 | 26.2 | 45.4 | 86.6 |
| 0.07 | - | - | - | - | - | - | 101.8 |

Correlation coefficients of the linear regressions were higher than 0.9945

**Table S3** Kinetic results for reaction of 2,6-bis(trifluoromethanesulfonyl)-4-nitroanisole with 4-methylaniline in MeOH-Me_2_SO (v/v) mixtures at 25 °C.

| [4-methylaniline]  / mol dm^-3^ | 10^4^ x k_obs_ / s^-1^ | | | | | |  |
| --- | --- | --- | --- | --- | --- | --- | --- |
|  | MeOH | MeOH-Me_2_SO (v/v) mixtures | | | | | Me_2_SO |
|  |  | 90-10 | 70-30 | 50-50 | 30-70 | 10-90 |  |
| 0.01 | 0.98 | 0.99 | 1.45 | 1.71 | 3.06 | 3.28 | 6.55 |
| 0.02 | 1.81 | 2.17 | 2.71 | 2.93 | 3.65 | 8.28 | 13.3 |
| 0.03 | 2.74 | - | - | - | - | - | - |
| 0.04 | - | 3.95 | 5.74 | 7.25 | 7.50 | 16.0 | 23.3 |
| 0.05 | 4.43 | - | - | - | - | - | 31.7 |
| 0.06 | - | 5.55 | 8.82 | 9.75 | 14.5 | 22.5 | 38.4 |
| 0.07 | 5.96 | - | - | - | - | - | 39.1 |
| 0.08 | - | 7.76 | 11.7 | 14.1 | 16.5 | 26.8 | 45.2 |
| 0.1 | 8.31 | 10.1 | 14.0 | 16.2 | 21.9 | 37.1 | 61.7 |

Correlation coefficients of the linear regressions were higher than 0.9903

**Table S4** Kinetic results for reaction of 2,6-bis(trifluoromethanesulfonyl)-4-nitroanisole with aniline in MeOH-Me_2_SO (v/v) mixtures at 25 °C.

| [aniline]  / mol dm^-3^ | 10^4^ x k_obs_ / s^-1^ | | | | | |  |
| --- | --- | --- | --- | --- | --- | --- | --- |
|  | MeOH | MeOH-Me_2_SO (v/v) mixtures | | | | | Me_2_SO |
|  |  | 90-10 | 70-30 | 50-50 | 30-70 | 10-90 |  |
| 0.01 | 0.62 | 0.65 | 0.71 | 1.02 | 1.40 | 2.38 | 3.97 |
| 0.02 | 1.09 | 1.15 | 1.47 | 2.07 | 2.40 | 4.66 | 6.84 |
| 0.03 | 1.58 | - | - | - | - | - | - |
| 0.04 | - | 2.12 | 2.75 | 4.13 | 4.70 | 8.82 | 13.4 |
| 0.05 | 2.55 | - | - | - | - | - | 16.6 |
| 0.06 | - | 3.36 | 4.02 | 5.81 | 7.37 | 12.4 | 19.2 |
| 0.07 | 3.86 | - | - | - | - | - | 21.5 |
| 0.08 | - | 4.25 | 5.63 | 8.26 | 9.80 | 17.4 | 27.1 |
| 0.1 | 5.00 | 5.55 | 6.92 | 10.1 | 12.1 | 23.1 | - |

Correlation coefficients of the linear regressions were higher than 0.9959

**Table S5** Kinetic results for reaction of 2,6-bis(trifluoromethanesulfonyl)-4-nitroanisole with 4-fluoroaniline in MeOH-Me_2_SO (v/v) mixtures at 25 °C.

| [4-fluoroaniline]  / mol dm^-3^ | 10^4^ x k_obs_ / s^-1^ | | | | | |  |
| --- | --- | --- | --- | --- | --- | --- | --- |
|  | MeOH | MeOH-Me_2_SO (v/v) mixtures | | | | | Me_2_SO |
|  |  | 90-10 | 70-30 | 50-50 | 30-70 | 10-90 |  |
| 0.005 | 0.32 | - | - | - | - | - | - |
| 0.01 | 0.48 | 0.49 | 0.68 | 1.07 | 1.09 | 2.55 | 3.49 |
| 0.02 | 0.90 | 1.19 | 1.34 | 1.75 | 2.61 | 3.68 | 4.66 |
| 0.025 | 1.19 | - | - | - | - | - | - |
| 0.03 | 1.53 | 1.60 | 2.01 | 2.85 | 3.40 | 6.78 | 7.92 |
| 0.04 | 1.89 | 2.13 | 2.60 | 3.83 | 4.37 | 9.33 | 10.1 |
| 0.05 | - | 2.59 | 3.55 | 4.75 | 5.30 | 10.6 | 13.8 |
| 0.06 | - | 3.23 | 3.90 | 5.66 | 7.03 | 12.9 | 17.0 |
| 0.07 | - | - | - | - | - | - | 19.2 |

Correlation coefficients of the linear regressions were higher than 0.9921

**Table S6** Kinetic results for reaction of 2,6-bis(trifluoromethanesulfonyl)-4-nitroanisole with 4-iodoaniline in MeOH-Me_2_SO (v/v) mixtures at 25 °C.

| [4-iodoaniline]  / mol dm^-3^ | 10^4^ x k_obs_ / s^-1^ | | | | | |  |
| --- | --- | --- | --- | --- | --- | --- | --- |
|  | MeOH | MeOH-Me_2_SO (v/v) mixtures | | | | | Me_2_SO |
|  |  | 90-10 | 70-30 | 50-50 | 30-70 | 10-90 |  |
| 0.01 | 0.25 | 0.29 | 0.39 | 0.51 | 0.60 | 0.88 | 1.48 |
| 0.02 | 0.51 | 0.67 | 0.71 | 0.94 | 1.32 | 2.39 | 3.51 |
| 0.04 | 0.98 | 1.19 | 1.41 | 2.14 | 2.79 | 5.23 | 5.30 |
| 0.05 | 1.25 | - | - | - | - | - | 7.74 |
| 0.06 | 1.58 | 1.74 | 2.22 | 3.22 | 3.72 | 6.76 | 8.81 |
| 0.07 | 1.89 | - | - | - | - | - | 10.9 |
| 0.08 | 2.11 | 2.56 | 2.98 | 4.20 | 5.38 | 9.97 | 12.5 |
| 0.1 | 2.57 | 3.07 | 3.74 | 5.02 | 6.40 | 10.7 | 14.8 |

Correlation coefficients of the linear regressions were higher than 0.9954

**Table S7** Kinetic results for reaction of 2,6-bis(trifluoromethanesulfonyl)-4-nitroanisole with 4-chloroaniline in MeOH-Me_2_SO (v/v) mixtures at 25 °C.

| [4-chloroaniline]  / mol dm^-3^ | 10^4^ x k_obs_ / s^-1^ | | | | | |  |
| --- | --- | --- | --- | --- | --- | --- | --- |
|  | MeOH | MeOH-Me_2_SO (v/v) mixtures | | | | | Me_2_SO |
|  |  | 90-10 | 70-30 | 50-50 | 30-70 | 10-90 |  |
| 0.01 | 0.27 | 0.28 | 0.32 | 0.43 | 0.55 | 0.85 | 1.48 |
| 0.02 | 0.38 | 0.49 | 0.61 | 0.75 | 1.12 | 2.09 | 2.51 |
| 0.03 | 0.55 | - | - | - | - | - | - |
| 0.04 | - | 1.16 | 1.25 | 1.84 | 2.35 | 4.44 | 5.30 |
| 0.05 | 0.94 | - | - | - | - | - | 6.74 |
| 0.06 | - | 1.25 | 1.82 | 2.24 | 2.85 | 5.88 | 7.07 |
| 0.07 | 1.38 | - | - | - | - | - | 9.06 |
| 0.08 | - | 1.85 | 2.48 | 3.49 | 4.60 | 8.55 | 10.5 |
| 0.1 | 2.07 | 2.58 | 3.05 | 4.0 | 5.34 | 9.98 | 12.9 |

Correlation coefficients of the linear regressions were higher than 0.9923

**Fig. S1** Effect of the p-hydroxyaniline concentration on the observed first-order rate constants (k_obs_) for addition of 2,6-bis(trifluoromethanesulfonyl)-4-nitroanisole at 25 °C in MeOH-Me_2_SO (v/v) mixtures.

**Fig. S2** Effect of the p-methoxyaniline concentration on the observed first-order rate constants (k_obs_) for addition of 2,6-bis(trifluoromethanesulfonyl)-4-nitroanisole at 25 °C in MeOH-Me_2_SO (v/v) mixtures.

**Fig. S3** Effect of the p-methylaniline concentration on the observed first-order rate constants (k_obs_) for addition of 2,6-bis(trifluoromethanesulfonyl)-4-nitroanisole at 25 °C in MeOH-Me_2_SO (v/v) mixtures.

**Fig. S4** Effect of the Aniline concentration on the observed first-order rate constants (k_obs_) for addition of 2,6-bis(trifluoromethanesulfonyl)-4-nitroanisole at 25 °C in MeOH-Me_2_SO (v/v) mixtures.

**Fig. S5** Effect of the p-fluoroaniline concentration on the observed first-order rate constants (k_obs_) for addition of 2,6-bis(trifluoromethanesulfonyl)-4-nitroanisole at 25 °C in MeOH-Me_2_SO (v/v) mixtures.

**Fig. S6** Effect of the p-iodoaniline concentration on the observed first-order rate constants (k_obs_) for addition of 2,6-bis(trifluoromethanesulfonyl)-4-nitroanisole at 25 °C in MeOH-Me_2_SO (v/v) mixtures.

**Fig. S7** Effect of the p-chloroaniline concentration on the observed first-order rate constants (k_obs_) for addition of 2,6-bis(trifluoromethanesulfonyl)-4-nitroanisole at 25 °C in MeOH-Me_2_SO (v/v) mixtures.
